# Hindlimb torsional alignment changes in growing rabbits after patellar dislocation 

**DOI:** 10.1186/s12891-021-03977-4

**Published:** 2021-01-29

**Authors:** Jinghui Niu, Qi Qi, Kang Piao, Kuo Hao, Iftekhar Sharif, Fei Wang

**Affiliations:** 1 Department of joint surgery, Hebei Medical University Third Affiliated Hospital, Shijiazhaung City, Hebei Province China; 2 Department of Cardiology, Hebei Medical University Third Affiliated Hospital, Shijiazhaung City, Hebei Province China

**Keywords:** Rabbits, Patellar dislocation, Growing, Bone malalignment

## Abstract

**Background:**

Torsional malalignment has been considered as a risk factor for patellar dislocation. But the influence of patellar dislocation for torsional alignment development remains unknown. The present study aims to investigate whether the torsional alteration of the hindlimb occurs after patellar dislocation in growing rabbits.

**Methods:**

In the present study, 30 one-month-old rabbits were included. The experimental group consisted of 30 left knees of rabbits which underwent patellar lateral dislocation. The control group consisted of 30 right knees of the rabbits which no surgical procedure was performed. The Computed Tomography (CT) scan was performed after the surgery and at the point the rabbits were skeletal mature (5 months post-surgery). The angles of femoral version and tibial torsion were measured using a three-dimensional method and analyzed between the experimental group and the control group.

**Results:**

After the surgery, the femoral version and tibial torsion in the experimental and control group were not significantly different. However, 5 months after surgery, the angle of femoral version in the experimental group (-5.50 ± 6.13°) was significantly different from that in the control group (−10.90 ± 4.74°) (*P* < 0.05). But the angle of tibial torsion in the experimental group (7.17 ± 7.25°) and control group (4.47 ± 6.34°) were not significantly different (*P* = 0.144).

**Conclusions:**

From this study, patellar dislocation can lead to alteration of femoral version in growing rabbits. So patellar dislocation may affect on lower extremity alignment. These findings may develop pathology and etiology of patellar dislocation.

## Background

Several anatomic factors are associated with patellar dislocation, including increased tibial tubercle–trochlear groove (TT-TG) distance, patella alta, rotational deformities, trochlear dysplasia,and patella shape [1–6]. Among the factors, rotational malalignment has been regarded as a risk factor for patellar dislocation in previous studies [2–4]. For femoral anteversion, Dejour et al. [2] found that the average femoral anteversion of normal knees was 10.8°, but the average femoral anteversion of knees with patellar instability was 15.6° (*P* = 0.013). Erkocak [3] and Takagi [4] also found the patients with a history of patellar instability had a higher mean femoral anteversion, compared with the normal’s. For tibial torsion, no significant difference was found between patients with patellofemoral instability and the normal [3, 5]. Clinically, strategic choice of surgical treatment for patients with patellar instability may be influenced by the torsion of the lower limbs [7]. For patients with patellar instability who have a femoral anteversion higher than 25°, single patellofemoral ligament reconstruction for patellar instability may be insufficient, and derotational femoral osteotomy should be considered [7].

Previous studies have investigated the influence of patellar dislocation on the development of the patellofemoral joint by rabbit models [8–10]. Wang [8], Li [9], and Niu [10] found femoral trochlear dysplasia and patellar dysplasia could occur after patellar dislocation in growing rabbits. On the other hand, Kaymaz found trochlea flattening after surgery for creating patella alta in growing rabbits [11]. These studies indicated that the dysplasia of patella or femoral trochlea could be caused by an abnormal patellar position. In another animal study [12], tibial tubercle lateralization and tibial tuberosity–trochlear groove distance (TT-TG) increased after patellar dislocation.

Although low extremity malalignment is considered as a predisposing factor for patellar dislocation [2–4], the effect of patellar dislocation on low extremity alignment development has remained unclear. To our best knowledge, this is the first study focusing on the alteration of the torsional alignment after the patellar dislocation. The objectives of the present study were to elucidate the alignment alteration in the transverse plane after patellar dislocation in growing rabbits and discuss the influence of patellar dislocation on lower extremity alignment. Based on the previous animal studies, we used the rabbit model for patellar dislocation and set the null hypothesis as that early patellar dislocation could not lead to a significant difference of torsional hindlimb alignment in growing rabbits.

## Methods

### Study Design and setting

This study was approved by the Animal Ethics Committee of the third hospital of Hebei Medical University (Number: Z2019-006-1; Date:2019-02-25). Sixty knees from 30 healthy, 1-month-old female New Zealand white rabbits, weighing between 350 and 450 g (provided by the Animal Center of the Hebei Medical University), were split into two groups. The experimental group consisted of 30 left knees, which were performed patellar dislocation surgery. The control group comprised 30 right knees with no surgical procedure. All procedures performed in studies involving animals were under the Western University’s Animal Care and Use Guidelines ( London, Ontario, Canada) [13].

The rabbits were kept individually in cages (310 × 550 × 320 mm), under controlled temperature (22 ± 2˚C), humidity (55 ± 5 %), 12-hour light-dark cycle (7:00 a.m. to 7:00 p.m.). The rabbits were raised with unrestricted access to standard water and food and were allowed 30 minutes of activity out of cages per day. The rabbits were euthanized by excessive anesthesia of pentobarbital sodium by injection through the ear vein at the end of the study.

### Surgical procedures

**Fig. 1:**
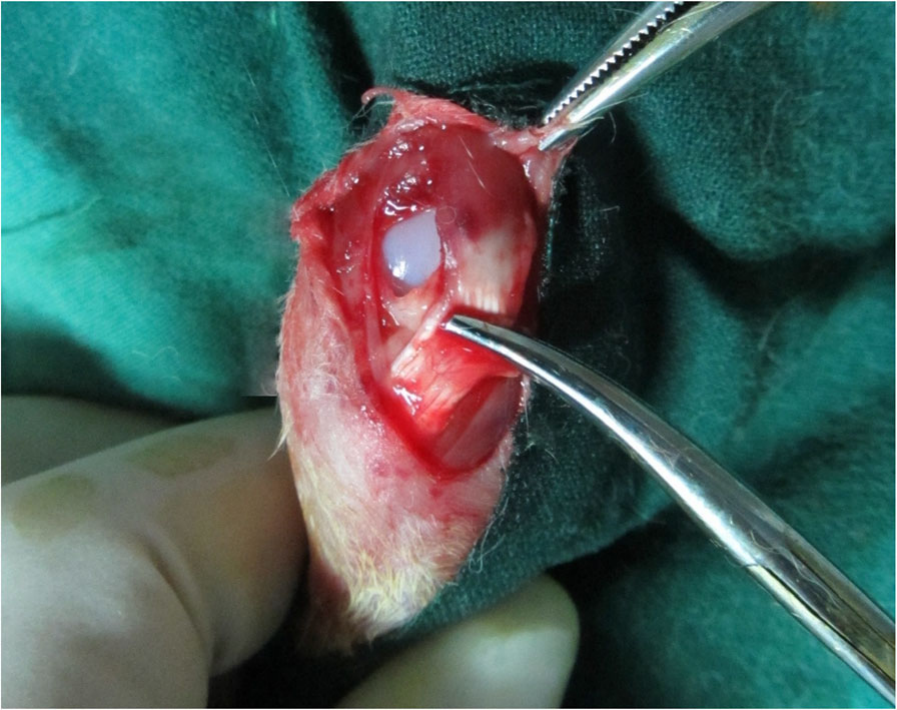
The picture during surgery. The medial retinaculum and joint capsule were incised. Patella was moved laterally and femoral trochlear could be seen

The processes for making patellar dislocation models of growing rabbits have been described and proved by previous studies [8–10]. The one-month-old rabbits were administered anesthesia of ketamine hydrochloride and xylazine at a dosage of 20 and 5 mg/kg body weight by injection through the ear vein. The rabbits were fixed on the platform for surgery with spine position. Then, the left knees of the experimental group were shaved and disinfected by standard procedures. A 2.5-cm incision was performed on the middle line of the knee skin, and the soft tissue was dissected to expose the medial retinaculum and the joint capsule. The medial retinaculum and medial joint capsule of the knees were incised about 1.5-cm, and the patella was then pushed laterally with hemostatic forcep to expose the femoral trochlea. At this time, the later joint capsule and lateral retinaculum were overlapped and sutured together. After these procedures, patellar dislocation could be seen intraoperatively (Fig. 1). The patella dislocated (the femoral trochlea was exposed) when the knee was flexed and extended. All the procedures were performed carefully to avoid cartilage and blood vessel damage. At last, the incision was interrupted sutured, and bandages were applied over the incision. CT scans were performed immediately after surgery to confirm lateral patellar dislocation. Ciprofloxacin (10 mg/kg, PO) was administered three days postoperatively for prophylaxis. The sutures were taken out two weeks postoperatively. The growing rabbits achieve skeletal maturation at 6 months [14], so the rabbits were euthanized at 5 months post-surgery when they were skeletally mature after the last follow-up.

### CT assessment

CT scans of the rabbits were performed immediately after the operation and 5 months postoperatively using a 16-slice CT scanner (SOMATOM Sensation 16; Siemens Medical Solutions, Erlangen, Germany). The rabbits were under anesthesia and were placed in a supine position. The hindlimb was fully extended and was fixed on a board to prevent any movements during scanning. Contiguous slices (1.0 mm) were obtained from the upper rim of the pelvis to the most distal part of the hindlimb. Considering the different structures of the hindlimb in rabbits and the accuracy of the measurements, the measurements were performed in a 3-dimensional strategy as the previous studies showed [4, 15–19]. The CT slices were sent to RadiAnt DICOM software (Medixant Ltd., Poznan, Poland) and reconstructed for 3D models. Our measuring methods had an accuracy of 0.01°.

**Fig. 2:**
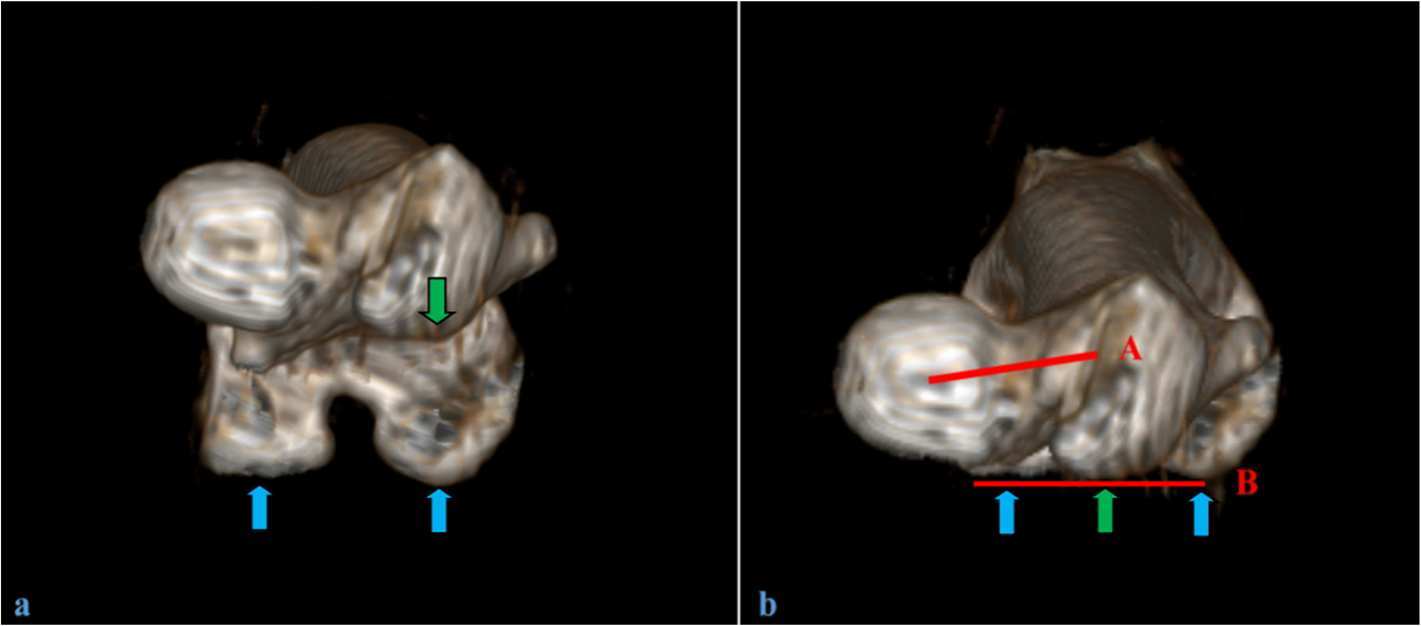
The schematic diagram of femoral version angle measurement. **a**: The superior view of the femur after 3D reconstruction. **b**: The 3D femur after adjustment. Line B connects the lowest point of the greater trochanter and femoral medial and lateral condyle. Line A connects the point of the center of the femoral head with the midpoint of the narrowest femoral neck. The femoral version was measured by the angle between Line A and Line B. The green arrow indicates the lowest point of the great trochanter. The blue arrows indicate the lowest point of the femoral medial and lateral condyle

The femoral version was measured as the study by Jia showed [15]. After 3D image construction, the femur was given a superior view (Fig. 2a). The lowest point of the greater trochanter, the femoral medial condyle, and femoral lateral condyle were moved and rotated for adjustment until the lowest point of the greater trochanter were positioned in the middle between the medial and lateral femoral condyle. The three points were connected by horizontal line B. The femoral neck version was the angle formed by the line B and line A which connecting the point of the femoral head center with the midpoint of the narrowest femoral neck part (Fig. 2b). The condition that the femoral neck is anterior to the posterior condylar line is defined as positive.

**Fig. 3:**
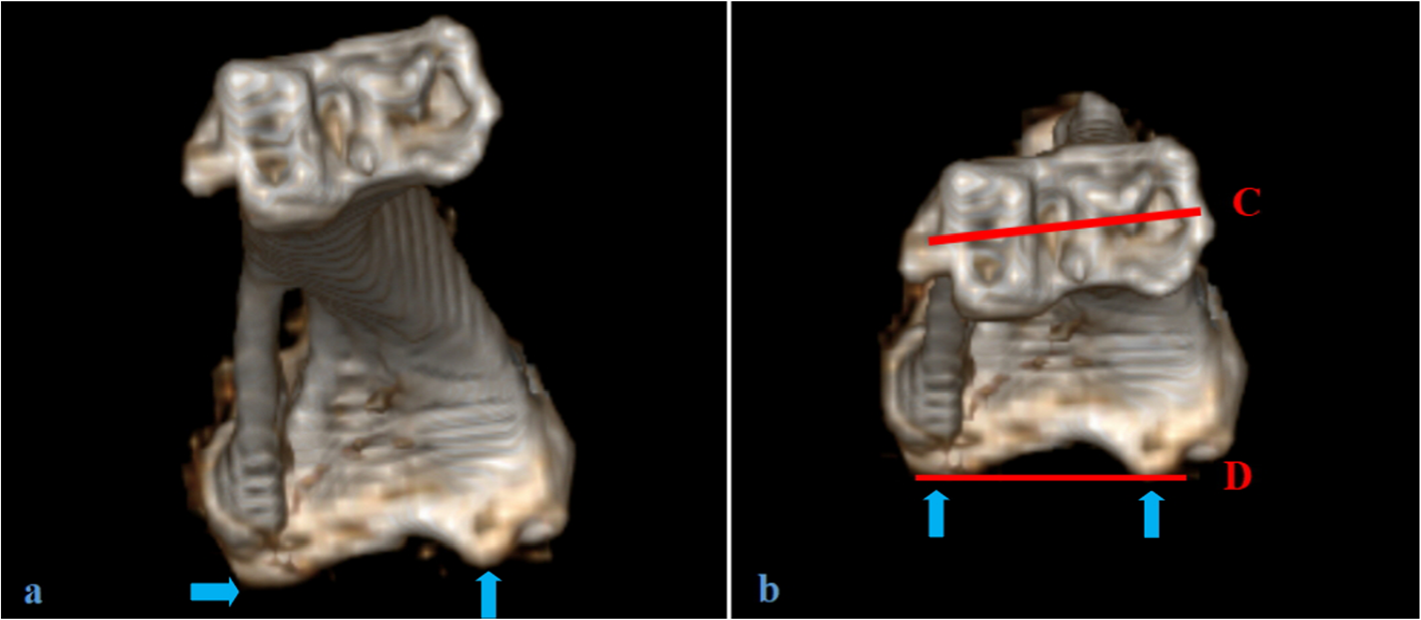
The schematic diagram of tibial torsion angle measurement. **a**: The inferior view of the tibia after 3D reconstruction. **b**: The 3D tibia after adjustment. Line D connects the medial and lateral tibial condyles. Line C is drawn through the center of medial and lateral malleoli. The tibial torsion was measured by the angle between Line C and Line D. The blue arrows indicate the lowest point of tibial medial and lateral condyle

For tibial torsion measurement, as in the previous study [4], the tibia was given an inferior view after 3D image reconstruction. (Fig. 3a). The tibia was rotated and moved until the tibia shaft was almost covered by the ankle joint. The most posterior points of the medial and lateral tibia condyle were connected by Line D. Tibial torsion was measured by the angle between the line D and the line C which was drawn through the center of lateral and medial malleoli (Fig. 3b). The condition that the ankle laterally rotated to the posterior tibial plateau is defined as positive.

### Statistics

Statistical analysis was performed using SPSS version 21.0 (SPSS, IL, USA). The results are expressed as mean ± standard deviation. Levene’s test was used to evaluate the homogeneity of the data. The mean difference of femoral version and tibial torsion between the control group and the experimental group were evaluated by Student’s *t* test. A *P* value < 0.05 was determined as statistically significant.

For determining the intra-observer variation, observer One(QQ) repeated the observations 2 weeks after the first measurement. To determine the inter-observer variation, the observations were performed by observer One(QQ), observer Two(KP), and observer Three(KH). The observers were blinded to the grouping. Intra-observer consistency and inter-observer consistency were analyzed using the intra-class correlation coefficient (ICC). ICC higher than 0.75 was excellent, ICC lower than 0.40 was poor, ICC among 0.40 to 0.75 was fair to good [20].

Based on the previous study [4, 21], the femoral version was selected as the primary variable for sample size calculation, the difference was set as 5° between the two groups, the standard deviation was assumed as 6°. With a confidence level of 95 % (α = 0.05) and power (1-β) of 80 %, the power calculation was performed and 24 knees were needed per group.

## Results

From CT scans, all the patellas dislocated laterally from femoral trochlea, which showed the patella dislocation model was achieved successfully. In this study, the femoral version and tibial torsion were measured immediately after surgery, and the values were not significantly different between the experimental group and the control group (Table 1). Two rabbits died of postoperative infection in one week after surgery. So 28 rabbits were taken CT scanning 5 months after surgery. The average weight of the 1-month-old rabbits was 0.4 kg, and at the last follow-up, the average weight was 2.8 kg. At that time, the femoral version of the experimental group was significantly different from that of the control group. But the tibial torsion in the experimental group and control group were not significantly different (Table 2). The intra-observer consistency and interobserver consistency were showed in Table 3.

**Table 1:** Measurements immediately after operation

Measurement(°)	Experimental group	Control group	N	*P* value
Femoral version	11.88 ± 4.89	13.50 ± 5.51	30	0.205
Tibia torsion	11.56 ± 4.03	12.94 ± 3.48	30	0.164

Significantly different: *P* < 0.05. Femoral version: positive: femoral neck is anterior to posterior condylar line; negative: femoral neck is posterior to posterior condylar line. Tibia torsion: positive: ankle laterally rotated to the posterior tibial plateau; negative: ankle internally rotated to the posterior tibial plateau.

**Table 2:** Measurements five months after operation

Measurement(°)	Experimental group	Control group	N	*P* value
Femoral version	−5.50 ± 6.13	−10.90 ± 4.74	28	0.001
Tibia torsion	7.17 ± 7.25	4.47 ± 6.34	28	0.144

Significantly different: *P* < 0.05. Femoral version: positive: femoral neck is anterior to posterior condylar line; negative: femoral neck is posterior to posterior condylar line. Tibia torsion: positive: ankle laterally rotated to the posterior tibial plateau; negative: ankle internally rotated to the posterior tibial plateau.

**Table 3:** Inter- and Intraobserver Reliability of the Different Measurements

	Intraclass Correlation Coefficient (95 % CI)		
Measurements		Immediately after surgery	5 months postoperatively
Femoral version	Intra-observer Reliability	0.931 (0.877–0.961)	0.939 (0.860–0.970)
	Inter-observer Reliability	0.912 (0.868–0.944)	0.870 (0.807–0.917)
Tibia torsion	Intra-observer Reliability	0.838 (0.733–0.902)	0.971 (0.953–0.984**)**
	Inter-observer Reliability	0.838 (0.765–0.893)	0.966 (0.947–0.979)

## Discussion

The most important finding of this study was that the femoral version was significantly different between the experimental group and control group in growing rabbits after patellar dislocation, and the tibial torsion was not significantly different between the two groups.

The femoral version shows the relative position of the femoral neck and the transcondylar axis or coronal plane of the distal femur. Femoral anteversion refers to the anterior rotation of the femoral head from the coronal plane of the femur. And femoral retroversion is defined as the condition that the femoral neck axis locates posterior to the transcondylar axis or the coronal plane of the femur [22].

For human beings, there is 30° to 40° of the femoral anteversion at birth. It decreases to 10° to 15° when skeletally mature. Most of the alteration occurs before the age of 8 years [23, 24]. For rabbits, there were 10° of anteversion in the femur at first. The anteversion disappeared by the eighth week, and by the time the rabbits were skeletally mature, 10° to 15° degrees of femoral retroversion has been observed [21]. Although the decreasing trends of the femoral version development between human-beings and rabbits may look numerically similar, the femoral version between them was different. Actually, for adults, femoral retroversion is not as common as femoral anteversion. In the study by Hartel, 1070 left femurs were performed thin-slice CT scans, and 77 subjects (7.8 %) were found with the retroverted femur (range − 23.6° – 0.2°) [25].

The femoral version relates to the stability and function of the knee and hip joints. The abnormal femoral version affects many diseases, including torsional syndromes, fractures of the femur, hip dysplasia, Legg-Calve-Perthes disease, and anterior cruciate ligament (ACL) rupture [26–30]. The femoral version also affects patellar stability. The increased femoral anteversion has been regarded as a risk factor for patellar instability, as it produces a lateralizing force on the patella [31]. The lateralizing force exists even after medial patellofemoral ligament reconstruction, contributes to the inferior clinical outcomes, even reconstruction failure [32, 33].

In this study, the femoral retroversion decreased after patellar dislocation in growing rabbits. Patellar dislocation may cause the alteration of the force direction of rectus femoris muscle. Also, we found knee or ankle lateral rotation in activities of rabbits after a patellar dislocation. The alteration of strength direction and the bone position may be the reason for the femoral version difference during growth. The version of the femur changed significantly after patellar dislocation, but the tibial torsion did not change significantly in the growing rabbits after patellar dislocation. Similar to humans, in the lower extremity, the femur may be abnormal although the tibia and fibula are well-formed or only slightly hypoplastic. And the foot may be normal despite the severe anomalies in the proximal part of the lower extremity [34].

For the previous experimental studies using rabbit models [8–10, 12], patellar dislocation or instability could lead to femoral dysplasia, patellar dysplasia, and higher TT-TG. A higher sulcus angle and lower trochlear depth, which indicated femoral trochlear dysplasia, were found in the rabbit knees after early patellar dislocation or instability [8, 9]. In another study, a longer diameter and higher Wiberg-angle of the patellas were found after patellar instability, which showed patellar dysplasia could occur after early patellar instability [10]. In the study by Niu, the TT-TG in the patellar dislocation group at the last follow-up was 3.0 ± 0.7 mm, while the TT-TG in the control group was 1.0 ± 0.4 mm (*P* < 0.05).

The femoral dysplasia, patellar dysplasia, and higher TT-TG were also regarded as risk factors for patellar dislocation [1–5]. So femoral dysplasia, patellar dysplasia, and high TT-TG are not only risk factors for patellar dislocation but also could be the consequences of patellar dislocation. In the present study, the aberrant femoral version was observed after patellar dislocation in the growing rabbits. Similarly, the abnormal femoral version may not only be a risk factor for patellar dislocation but also be the consequence of patellar dislocation. These findings may develop pathology and etiology of patellar instability, and emphasize the importance of the early effective treatments for patellar instability in children, considering the possibility of pathological conditions caused by femoral version deformity.

From previous studies, patellar dislocation has been successfully achieved in growing rabbits after patellar dislocation surgery [8–10, 12]. In the present studies, patellar dislocation was observed by CT scans from each rabbit immediately after surgery and at the last follow-up, which showed the patellar dislocation model was obtained successfully. It is possible that alignment values have not been measured precisely because two-dimensional (2D) measurements can be affected by the location of the radiation source and the limb position [4]. Recently, a three-dimensional (3D) method for measuring the alignment of the lower extremity has been widely used, which was proved to have high intra-observer and inter-observer reliability. And the method is not influenced by the femoral neck-shaft angle or postural deformity [4, 15–18]. Considering the high accuracy of the method and the extreme flexion of the knee and hip joints in rabbits, the 3D method has been taken into account in this study and achieved high intra-observer and inter-observer reliability (Table. 3).

The are several limitations of the study. First, the structure of the hindlimbs of rabbits is different from human beings’. For example, adults often have femoral anteversion while mature rabbits often have femoral retroversion. On the other hand, although the rabbits had patellar dislocation surgery at one-month-old to imitate the early patellar dislocation of humans, it still could not imitate the first patellar dislocation of humans for they have abnormal knees presumably due to a genetic abnormality. So the conclusion of this study may not apply to humans. But the rabbit models have been widely used for patellar dislocation studies [8–10, 12]. The present study is the first research focusing on the influence of patellar dislocation to torsional alignment. It has high intra-observer and inter-observer reliability, which may enrich the etiology and pathology of patellar dislocation. Second, the knee rotation measurements were not involved in this study because of the extreme flexion in the knee joints in the rabbits. But the femoral version and tibial torsion can sufficiently reflect the torsional alignment of rabbit hindlimbs. The third limitation is the sample size of the rabbits. Although the sample size is enough according to the sample size calculation, it could be more reliable if a higher number of experimental animals were used. Also, the reason for the alteration of the femoral version at the biomechanical and molecular level should be researched in the future.

## Conclusions

Based on the outcomes of this study, we conclude that early patellar dislocation can lead to the abnormal femoral version in growing rabbits. So patellar dislocation may affect lower extremity alignment.

## Data Availability

The detailed data and materials of this study are available from the corresponding author via e-mail on reasonable request..
